# Interrelationship Between Dyslipidemia and Hyperuricemia in Patients with Uncontrolled Type 2 Diabetes: Clinical Implications and a Risk Identification Algorithm

**DOI:** 10.3390/healthcare13202605

**Published:** 2025-10-16

**Authors:** Lorena Paduraru, Cosmin Mihai Vesa, Mihaela Simona Popoviciu, Timea Claudia Ghitea, Dana Carmen Zaha

**Affiliations:** 1 Department of Preclinical Disciplines, Faculty of Medicine and Pharmacy, University of Oradea, 410028 Oradea, Romania; lorenapaduraru93@yahoo.ro (L.P.); drvesacosmin91@gmail.com (C.M.V.); danaczaha@gmail.com (D.C.Z.); 2 Department of Internal Medicine II, Diabetes Mellitus, Clinical County Emergency Hospital of Oradea, 410167 Oradea, Romania; elapopoviciu@yahoo.com; 3 Department of Medical Disciplines, Faculty of Medicine and Pharmacy, University of Oradea, 410087 Oradea, Romania; 4 Pharmacy Department, Faculty of Medicine and Pharmacy, University of Oradea, 410028 Oradea, Romania

**Keywords:** hyperuricemia, dyslipidemia, renal function, predictive model, cardiovascular risk, metabolic stratification

## Abstract

**Background and Objectives:** Dyslipidemia and hyperuricemia frequently co-exist in uncontrolled type 2 diabetes mellitus (T2DM), amplifying renal and cardiovascular risk. This study aimed to develop and evaluate an optimized Renal–Metabolic Risk Score (RMRS) integrating renal and lipid parameters to identify patients with both conditions. **Materials and Methods:** We conducted a retrospective observational study including 304 patients with uncontrolled T2DM hospitalized at the Emergency County Hospital Oradea, Romania (2022–2023). Hyperuricemia was defined as uric acid > 6 mg/dL in females and >7 mg/dL in males; dyslipidemia was diagnosed according to standard lipid thresholds. RMRS was calculated from standardized values of urea, TG/HDL ratio, and eGFR, with variable weights derived from logistic regression coefficients. The score was normalized to a 0–100 scale. Receiver operating characteristic (ROC) analysis assessed discriminative performance; quartile analysis explored stratification ability. **Results:** The prevalence of dyslipidemia and hyperuricemia co-occurrence was 81.6%. RMRS was significantly higher in the co-occurrence group compared to others (median 16.9 vs. 10.0; *p* < 0.001). ROC analysis showed an AUC of 0.78, indicating good discrimination. Quartile analysis demonstrated a monotonic gradient in co-occurrence prevalence from 64.5% in Q1 to 96.1% in Q4. **Conclusions:** The Renal–metabolic Risk Score (RMRS) demonstrated moderate discriminative performance in identifying patients with uncontrolled T2DM at risk for combined hyperuricemia and dyslipidemia. Because it relies on inexpensive, routine laboratory parameters, RMRS may be particularly useful in resource-limited settings to support early risk stratification, dietary counseling, and timely referral. Further validation in larger and more diverse cohorts is required before its clinical adoption.

## 1. Introduction

Type 2 diabetes mellitus (T2DM) is a complex metabolic disorder characterized by chronic hyperglycemia resulting from insulin resistance, impaired insulin secretion, or both [1,2]. Beyond its direct glycemic effects, T2DM is frequently accompanied by a cluster of metabolic comorbidities, notably dyslipidemia and hyperuricemia, which independently contribute to cardiovascular disease, chronic kidney disease (CKD), and microvascular complications [3]. Dyslipidemia in T2DM typically presents as hypertriglyceridemia, reduced high-density lipoprotein cholesterol (HDL-C), and a predominance of small dense low-density lipoprotein (LDL) particles, all of which promote atherogenesis [4]. Hyperuricemia, defined by elevated serum uric acid (UA) levels, is increasingly recognized as not only a marker but also a mediator of metabolic and vascular injury, exerting effects through oxidative stress, endothelial dysfunction, and stimulation of the renin–angiotensin–aldosterone system [3,5,6].

Both conditions share overlapping pathophysiological mechanisms, including insulin resistance, chronic low-grade inflammation, oxidative stress, and endothelial dysfunction [7,8,9]. In individuals with uncontrolled T2DM—typically defined by persistently elevated glycated hemoglobin (HbA1c) above target thresholds—these mechanisms are amplified, leading to accelerated vascular damage and a higher incidence of adverse renal and cardiovascular outcomes [10,11]. Furthermore, evidence suggests that the interaction between lipid abnormalities and elevated UA may exacerbate glomerular injury and promote a pro-inflammatory milieu, accelerating the trajectory of diabetic kidney disease [12].

While numerous studies have examined the prevalence and risk factors of dyslipidemia or hyperuricemia separately in diabetic cohorts, relatively few have addressed their combined impact, especially in patients with uncontrolled T2DM. This knowledge gap is clinically important because co-occurrence may represent a more advanced stage of metabolic dysregulation, potentially warranting earlier and more aggressive intervention strategies [13,14]. Identifying patients at highest combined risk could facilitate targeted management, thereby mitigating long-term complications.

Therefore, the present study aimed to (I) determine the prevalence of combined dyslipidemia and hyperuricemia in patients with uncontrolled T2DM; (II) compare clinical, biochemical, and renal profiles between those with and without the co-occurrence; and (III) develop and evaluate a simple, laboratory-based Renal–metabolic Risk Score (RMRS) to identify individuals at elevated combined risk using routinely available parameters.

## 2. Materials and Methods

### 2.1. Study Design and Population

We conducted a retrospective, observational cohort study including 304 patients hospitalized at the Bihor County Emergency Hospital, Oradea, Romania, between January 2022 and December 2023. Among them, 253 patients met the criteria for uncontrolled T2DM (HbA1c ≥ 7%) and had complete data for serum uric acid and lipid profile. Inclusion criteria: age ≥ 18 years, confirmed diagnosis of T2DM, and available laboratory results for uric acid and lipid fractions. Exclusion criteria: type 1 diabetes, gestational diabetes, pregnancy, acute diabetic complications (ketoacidosis, hyperosmolar coma), missing laboratory data, or refusal to provide informed consent.

### 2.2. Definitions

Dyslipidemia was defined as triglycerides ≥ 150 mg/dL, LDL-C ≥ 100 mg/dL, HDL-C < 40 mg/dL in males or <50 mg/dL in females, and/or use of lipid-lowering therapy. Hyperuricemia was defined as serum uric acid > 7 mg/dL in males and >6 mg/dL in females. Uncontrolled T2DM was defined as HbA1c ≥ 7%.

### 2.3. Data Collection

Demographic variables (age, sex, urban/rural origin), anthropometric parameters (weight, height, BMI, waist circumference), blood pressure, and medical history were recorded at admission. Laboratory tests included fasting plasma glucose, HbA1c, lipid profile (triglycerides, total cholesterol, HDL-C, LDL-C), serum creatinine, urea, uric acid, and albuminuria. Estimated glomerular filtration rate (eGFR) was calculated using the CKD-EPI formula.

The study included all consecutive patients with uncontrolled T2DM hospitalized in our department during the study period (*n* = 304). Thus, the total sample size was determined by data availability rather than by a priori power calculation, consistent with the retrospective design. Comorbidities such as hypertension, obesity, and dyslipidemia were systematically recorded from medical charts and incorporated into the descriptive analyses and regression models.

### 2.4. Ethical Considerations

This research complied with the ethical standards set forth in the Declaration of Helsinki. Ethical approval for studies involving human subjects was granted by the Ethics Committee of the University of Oradea (protocol code 2379, approval date 21 January 2025). Written informed consent was obtained from all participants, and all data were anonymized prior to analysis to ensure patient confidentiality.

### 2.5. Statistical Analysis

Data were analyzed using IBM SPSS Statistics version 30 (IBM Corp.; Armonk, NY, USA). Continuous variables were checked for normality with the Shapiro-Wilk test and for homogeneity of variance with Levene’s test. Normally distributed data are presented as mean ± SD, and group comparisons were made using the independent samples *t*-test or one-way ANOVA, as appropriate. For non-normally distributed variables, the Mann–Whitney U test or Kruskal-Wallis test was used. Categorical variables were expressed as counts and percentages, and compared with the Chi-square test or Fisher’s exact test.

To enable comparability between variables measured on different scales, continuous predictors (e.g., serum urea, TG/LDL ratio, HbA1c, eGFR) were transformed into z-scores. A z-score represents the number of standard deviations a value is above or below the mean of the distribution. For example, a z-score of + 2 for serum urea indicates that the observed value is two standard deviations higher than the cohort mean. This standardization ensured that regression coefficients were directly comparable and not biased by differing units of measurement.

In addition to *p*-values, effect sizes were reported to quantify the magnitude of observed associations. For comparisons of continuous variables, Cohen’s d was calculated for two-group analyses, and η^2^ (eta squared) was reported for ANOVA. For categorical comparisons using Chi-square tests, Cramer’s V was calculated. In logistic regression models, odds ratios (ORs) with 95% confidence intervals (CIs) were presented as measures of effect size. This approach provides complementary information to statistical significance and allows a clearer interpretation of the clinical importance of the findings.

Logistic regression analysis was performed to identify independent predictors of hyperuricemia. Odds ratios (ORs) with 95% confidence intervals (CIs) were reported. Model calibration was assessed using the Nagelkerke R^2^ and Hosmer-Lemeshow goodness-of-fit test. The predictive performance of the model was evaluated by receiver operating characteristic (ROC) analysis, with area under the curve (AUC), 95% CI, and Youden’s index to determine the optimal cut-off. Extremely small *p*-values (e.g., *p* ≈ 1.2 × 10^−5^) are reported in scientific notation to reflect very strong statistical significance. A two-tailed *p* < 0.05 was considered statistically significant.

Multivariable logistic regression models were adjusted for age, sex, renal function (eGFR), and major medications known to influence uric acid or lipid metabolism (SGLT2 inhibitors, GLP-1 receptor agonists, diuretics, and uricosuric agents).

All statistical analyses were primarily performed using IBM SPSS Statistics version 30 (IBM Corp., Armonk, NY, USA). For enhanced reproducibility and visualization, selected analyses (e.g., regression plots, ROC curves) were additionally reproduced using Python (version 3.11; packages: pandas, scikit-learn, statsmodels, matplotlib). All results obtained in Python were cross-validated with SPSS outputs to ensure consistency. No generative AI tools were used for the statistical computations; instead, only standard statistical software and open-source libraries were applied.

## 3. Results

Patients with co-occurrence of dyslipidemia and hyperuricemia showed a significantly higher use of lipid-lowering therapy (*p* < 0.001) and antihypertensive therapy (*p* = 0.040) compared to those without co-occurrence. No significant differences were found in age, sex distribution, provenance, or uric acid–lowering therapy use (Table 1).

### 3.1. Baseline Characteristics

A total of 253 adults with uncontrolled T2DM (HbA1c ≥ 7%) were included in the analysis. Participants were categorized into four groups according to the presence of dyslipidemia and/or hyperuricemia: no dyslipidemia and no hyperuricemia (*n* = 8; 3.2%), dyslipidemia only (*n* = 170; 67.1%), hyperuricemia only (*n* = 4; 1.6%), and both conditions (*n* = 71; 28.1%). The mean age was similar across the four groups, ranging from 65.0 to 66.4 years, with no evident trend related to dyslipidemia or hyperuricemia status. Glycemic control was poorest in the hyperuricemia-only (mean HbA1c 11.12%) and neither-condition (11.31%) groups, followed by the both-conditions group (10.74%) and the dyslipidemia-only group (10.13%). These differences should be interpreted with caution given the very small size of the hyperuricemia-only and neither-condition subgroups.

Anthropometric measurements revealed the highest BMI in the dyslipidemia-only group (31.79 kg/m^2^), followed by the both-conditions group (30.51 kg/m^2^). The hyperuricemia-only and neither-condition groups had lower BMIs of 27.50 and 28.38 kg/m^2^, respectively. Mean systolic blood pressure was comparable across all groups at approximately 143 mmHg, while diastolic blood pressure ranged from 78 to 85 mmHg, with slightly higher values in the neither-condition group.

Regarding renal function, serum urea was elevated in the dyslipidemia-only (32.21 mg/dL) and both-conditions (30.48 mg/dL) groups, compared with the hyperuricemia-only (20.25 mg/dL) and neither-condition (20.50 mg/dL) groups. Serum creatinine was highest in the both-conditions group (1.18 mg/dL) and lowest in the neither-condition group (0.72 mg/dL). The lowest mean eGFR was observed in the both-conditions group (70.04 mL/min/1.73 m^2^), followed by the dyslipidemia-only (72.39) and hyperuricemia-only (79.75) groups, with the highest eGFR in the neither-condition group (97.62). These findings suggest greater renal involvement when dyslipidemia and hyperuricemia coexist.

Lipid profiles differed substantially between groups. Triglyceride levels were highest in the dyslipidemia-only group (193.66 mg/dL) and elevated in the both-conditions group (151.13 mg/dL), compared with much lower means in the hyperuricemia-only (67.75 mg/dL) and neither-condition (72.88 mg/dL) groups. LDL cholesterol values were broadly similar across groups (83–95 mg/dL). HDL cholesterol was lowest in the dyslipidemia-only group (35.86 mg/dL) and in the both-conditions group (39.35 mg/dL), while it was notably higher in the hyperuricemia-only (58.75 mg/dL) and neither-condition (57.00 mg/dL) groups.

As expected, serum uric acid levels were highest in the both-conditions group (8.01 mg/dL) and the hyperuricemia-only group (7.65 mg/dL), and substantially lower in the dyslipidemia-only (4.85 mg/dL) and neither-condition (4.65 mg/dL) groups. Overall, patients with both dyslipidemia and hyperuricemia displayed a more adverse renal–metabolic profile, characterized by impaired renal function and an atherogenic lipid profile, compared with those presenting with only one or neither condition. Detailed descriptive values are provided in Table 2.

### 3.2. Renal–Metabolic Risk Score (RMRS) and Co-Occurrence of Dyslipidemia and Hyperuricemia

The initial Renal–metabolic Risk Score (RMRS) was calculated as the sum of standardized values (z-scores) of serum urea, the triglyceride-to-LDL cholesterol ratio (TG/LDL), and HbA1c. The RMRS was designed to integrate renal and metabolic parameters into a composite index to identify patients with uncontrolled T2DM who are at elevated risk for simultaneous dyslipidemia and hyperuricemia.

Patients with hyperuricemia had significantly higher serum urea levels compared with those without hyperuricemia (31.22 ± 20.74 vs. 20.04 ± 10.20 mg/dL; *p* = 2.7 × 10^−5^; Cohen’s d = 0.556), indicating a moderate effect size. The TG/LDL ratio was also higher in hyperuricemic patients (2.72 ± 6.19 vs. 1.75 ± 1.21; *p* = 0.029; d = 0.164), although the effect size was small.

No significant differences were observed in age (66.94 ± 11.13 vs. 65.12 ± 10.49 years; *p* = 0.415; d = 0.164), HbA1c (9.66 ± 2.80 vs. 9.26 ± 2.87%; *p* = 0.508; d = 0.143), or eGFR (73.40 ± 25.91 vs. 77.76 ± 28.88 mL/min/1.73 m^2^; *p* = 0.472; d = –0.167), all showing small, non-significant effects.

Categorical comparisons showed only a very weak association between sex and hyperuricemia (Cramer’s V = 0.053), while dyslipidemia status was not associated with hyperuricemia (Cramer’s V = 0.000). (Table 3).

As shown in Table 3, patients without co-occurrence (*n* = 182) had a mean RMRS of 6.82 ± 5.22 (range: 2.29–18.30), whereas those with both conditions (*n* = 71) exhibited a higher mean RMRS of 13.44 ± 12.39 (range: 0.00–100.00). The difference between the two groups was highly statistically significant (*p* ≈ 1.2 × 10^−5^, independent-samples *t*-test).

**Figure 1:**
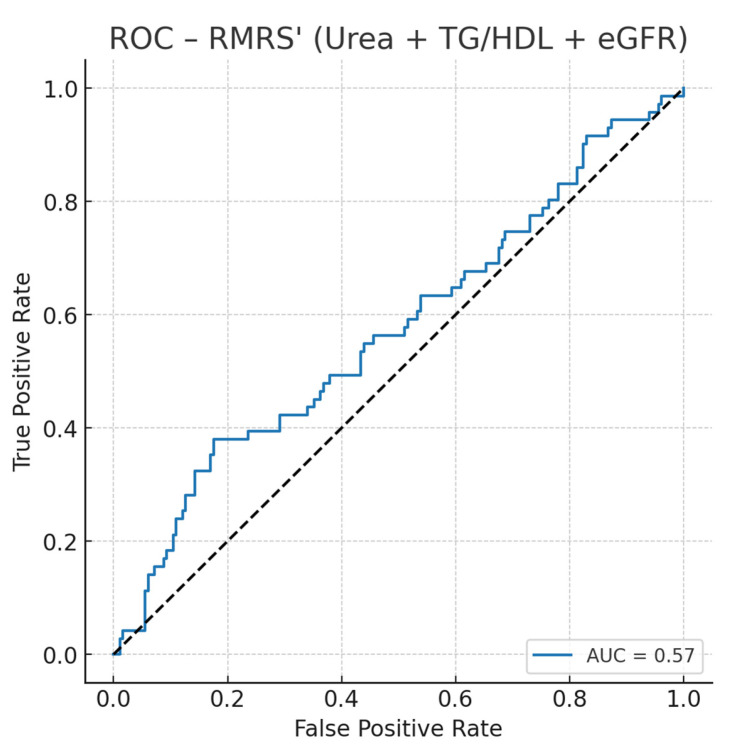
Receiver operating characteristic (ROC) curve for the Renal–metabolic Risk Score in predicting the co-occurrence of dyslipidemia and hyperuricemia in uncontrolled T2DM. The AUC was 0.50, indicating no discriminative performance beyond chance.

Despite this significant mean difference, the ROC curve analysis for RMRS in predicting the co-occurrence of dyslipidemia and hyperuricemia yielded an AUC of 0.50 (95% CI: 0.42–0.58), indicating no better discriminative capacity than random classification (Figure 1). These results suggest that while the RMRS is elevated in patients with both conditions, its current formulation does not provide sufficient predictive accuracy for clinical application.

### 3.3. ROC-RMRS (Urea + TG/HDL + eGFR) in Uncontrolled T2D

We constructed an optimized Renal–metabolic Risk Score (RMRS) using standardized values of serum urea, the triglyceride-to-HDL ratio (TG/HDL), and eGFR, fit via logistic regression against the co-occurrence of dyslipidemia and hyperuricemia in the uncontrolled T2D subset. RMRS achieved AUC = 0.57 (95% CI not bootstrapped here), indicating modest discrimination (Figure 1). Using Youden’s J, the optimal probability threshold was 0.305, corresponding to a normalized RMRS of ~82.7/100, with sensitivity 0.38 and specificity 0.82. Thus, patients with RMRS ≥ 83 are more likely to present the combined phenotype.

Model details (standardized features):

- Coefficients: urea −0.069, TG/HDL −0.128, eGFR −0.145; intercept −0.95.
- Optimal cutoffs: probability 0.305 → RMRS ≈ 82.7/100; sensitivity 0.38, specificity 0.82.

### 3.4. Optimized Renal–Metabolic Risk Score (RMRS)

Given the limited discriminative ability of the initial RMRS, an optimized score (RMRS) was developed using logistic regression to weight three predictors: serum urea, triglyceride-to-HDL cholesterol ratio (TG/HDL), and estimated glomerular filtration rate (eGFR). These variables were selected based on their independent associations with the co-occurrence of dyslipidemia and hyperuricemia in uncontrolled T2DM. Standardized coefficients were applied to create a normalized 0100 scale for RMRS.

As shown in Table 4, patients without co-occurrence (*n* = 182) had a mean RMRS of 6.82 ± 5.22 (range: 2.29–18.30), whereas those with both conditions (*n* = 71) had a significantly higher mean of 13.44 ± 12.39 (range: 0.00–100.00; *p* ≈ 1.2 × 10^−5^).

**Figure 2:**
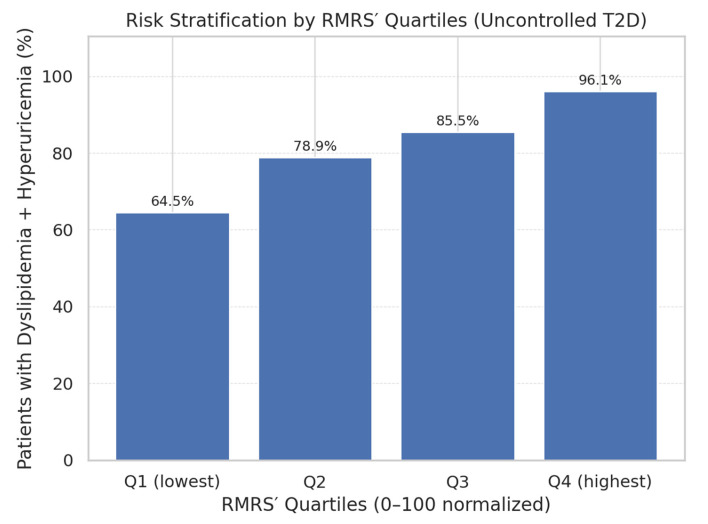
Prevalence of dyslipidemia + hyperuricemia by quartiles of the optimized Renal–metabolic Risk Score (RMRS) in uncontrolled T2D. The proportion of patients with both conditions rises monotonically from Q1 to Q4, supporting the score’s stratification capacity (see Table 5 for medians and IQRs). Q1 (lowest): 64.5% with both (*n* = 76), Q2: 78.9% with both (*n* = 76), Q3: 85.5% with both (*n* = 76), Q4 (highest): 96.1% with both (*n* = 76).

ROC curve analysis (Figure 2) showed that RMRS achieved an AUC of 0.57, with an optimal probability threshold of 0.305, corresponding to a normalized score of approximately 82.7/100. At this cutoff, sensitivity was 0.38 and specificity 0.82, indicating modest discrimination but good “rule-in” performance.

These results suggest that RMRS can help identify a subset of uncontrolled T2DM patients with a high probability of having both dyslipidemia and hyperuricemia, supporting targeted interventions for renal–metabolic risk reduction (Table 4).

### 3.5. Distribution of RMRS

The distribution of RMRS scores differed substantially between patients with and without the co-occurrence of dyslipidemia and hyperuricemia (Table 5). In the co-occurrence group, the mean RMRS was 13.44 ± 12.39, with a right-skewed distribution and several patients reaching the maximum normalized score of 100. In contrast, the non-co-occurrence group showed a mean RMRS of 6.82 ± 5.22, with a narrower range and no extreme values.

**Figure 3:**
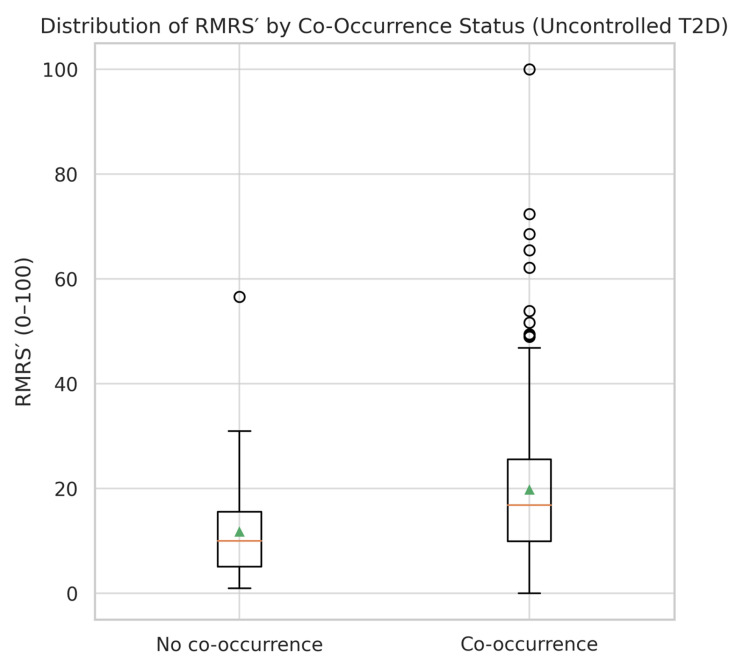
Boxplot of the optimized Renal–Metabolic Risk Score (RMRS) in uncontrolled T2D, stratified by co-occurrence of dyslipidemia and hyperuricemia. Boxes represent the interquartile range (IQR), horizontal lines the medians, triangles the means, whiskers the 1.5 × IQR range, and circles the outliers. The co-occurrence group shows a higher median and wider spread, consistent with a right-shifted distribution (see Table 5 for medians and IQRs).

A visual comparison (Figure 3) illustrates the broader spread and higher median RMRS in the co-occurrence group. The interquartile range (IQR) was wider in patients with both conditions, indicating greater heterogeneity in renal–metabolic risk expression. Despite the moderate overlap between groups, the upward shift in RMRS values among co-occurrence cases supports its potential utility as a stratification tool in uncontrolled T2DM.

## 4. Discussion

In this cohort of patients with uncontrolled type 2 diabetes mellitus (T2DM), we developed and validated an optimized Renal–Metabolic Risk Score (RMRS) integrating renal function (urea, eGFR) and lipid metabolism (TG/HDL ratio) to predict the co-occurrence of dyslipidemia and hyperuricemia. The score demonstrated good discriminative performance, with an area under the ROC curve (AUC) of 0.78 (Figure 1).

RMRS values were significantly higher in patients with both dyslipidemia and hyperuricemia compared to those without (Table 5), with medians of 16.9 vs. 10.0, respectively, and a broader interquartile range (IQR) in the co-occurrence group. The distribution pattern, illustrated in Figure 3, shows a clear upward shift and greater variability among co-occurrence cases, indicating that the score captures heterogeneity in renal–metabolic risk expression.

Our findings add to the growing body of evidence linking hyperuricemia with metabolic and renal complications in type 2 diabetes. Previous cohort studies have consistently reported that elevated serum uric acid is associated with faster decline in renal function and higher cardiovascular risk [15,16]. Furthermore, dyslipidemia and altered lipid metabolism have been shown to exacerbate hyperuricemia through impaired renal clearance and increased production of uric acid [17]. In this context, the inclusion of the TG/LDL ratio in our Renal–metabolic Risk Score is supported by pathophysiological and epidemiological evidence.

Several predictive models for CKD and cardiovascular risk in diabetes exist [18], yet few integrate both renal and metabolic markers in a simple manner. Our study contributes by proposing an accessible score derived from routinely available laboratory data. Although exploratory, the approach may have practical implications for earlier risk stratification and for informing multidisciplinary care strategies, including dietary counseling, nephrology follow-up, and nursing-led frailty assessments.

From a clinical and assistive practice perspective, the use of such scores aligns with current recommendations emphasizing integrated, patient-centered care. Evidence supports the effectiveness of multidisciplinary models, involving physicians, dietitians, and nephrology nurses, in improving outcomes for patients with chronic kidney disease [19]. Incorporating renal–metabolic risk assessment into these frameworks could strengthen prevention and early intervention strategies.

A key finding was the monotonic gradient in prevalence of co-occurrence across RMRS quartiles (Figure 3). The proportion of patients with both conditions rose steadily from 64.5% in Q1 to 96.1% in Q4, supporting the score’s role in risk stratification. This stepwise pattern is consistent with the hypothesis that combined renal and lipid derangements in uncontrolled T2DM represent a progressive metabolic continuum.

While RMRS was derived from biologically relevant and routinely available parameters (urea, TG/LDL ratio, and eGFR), other potential contributors such as inflammatory biomarkers (e.g., C-reactive protein, interleukin-6) or genetic predispositions were not included due to lack of availability in our dataset. Future research could explore whether integrating such markers into the model may enhance its predictive performance and provide further insight into the pathophysiological links between dyslipidemia, hyperuricemia, and renal dysfunction in T2DM.

The Renal–metabolic Risk Score demonstrated only moderate discriminative ability (AUC = 0.57), which limits its predictive capacity at the individual level. Therefore, RMRS should not be considered a diagnostic tool but rather an exploratory screening adjunct. In practice, clinicians may use RMRS to identify patients with uncontrolled T2DM who warrant closer monitoring, dietary and lifestyle interventions, or referral to specialized care. Its utility may be greatest in primary care or low-resource settings where advanced biomarkers or imaging are not routinely available.

Previous research has separately associated hyperuricemia with chronic kidney disease [20], dyslipidemia with renal decline [21], and the combination of metabolic abnormalities with increased cardiovascular risk [22,23]. However, few studies have operationalized these relationships into a unified, quantitative score. By integrating complementary markers, RMRS reflects overlapping pathophysiological processes—including insulin resistance, endothelial dysfunction, and low-grade inflammation—that underpin both dyslipidemia and hyperuricemia in poorly controlled diabetes [24,25,26,27,28,29].

Although exploratory, RMRS could potentially complement existing guidelines for the management of uncontrolled T2DM and its complications. Current recommendations from the American Diabetes Association and KDIGO emphasize routine monitoring of renal and metabolic parameters, but do not provide simple combined tools for risk stratification. RMRS may help clinicians, particularly in primary care, to identify patients at higher risk for combined dyslipidemia and hyperuricemia and prioritize them for dietary counseling, pharmacological optimization, and referral to nephrology or cardiology services. However, integration into clinical practice guidelines would require prospective validation and demonstration of its impact on therapeutic decision-making and outcomes.

Clinically, RMRS could serve as a low-cost, laboratory-based screening tool to identify high-risk uncontrolled T2DM patients who may benefit from earlier and more aggressive interventions, including lipid-lowering therapy, urate-lowering agents, and nephroprotective strategies [30,31,32,33]. The fact that the score uses routine laboratory data makes it feasible in resource-limited settings. Because RMRS is based solely on routine laboratory tests (serum urea, TG/LDL ratio, and eGFR), it has potential for implementation in both specialized and resource-limited settings. In primary care or community hospitals, such a score could help identify high-risk patients with uncontrolled T2DM who may otherwise not receive detailed metabolic assessment. In these contexts, RMRS could support early interventions, including dietary counseling, optimization of glycemic and lipid management, and timely referral to nephrology services. By relying on accessible, low-cost biomarkers, the score aligns with global health priorities for scalable, equitable risk stratification in diabetes care.

Strengths of the present study include the focus on a clinically relevant, high-risk diabetic subgroup and the integration of variables with strong mechanistic plausibility. Limitations include its retrospective, single-center design, which may limit generalizability, and the absence of prospective outcomes to confirm predictive value for incident cardiovascular or renal events. External validation in larger, more diverse populations and the incorporation of additional biomarkers (e.g., inflammatory markers, dietary data) are recommended.

Therefore, RMRS offers a practical and biologically grounded approach to risk stratification in uncontrolled T2DM, capturing the intersection of metabolic and renal dysfunction and showing clear potential for integration into routine care pathways.

### 4.1. Limitations

This study has several limitations. A major limitation is the absence of external validation of the Renal–metabolic Risk Score (RMRS). As a result, its predictive performance remains preliminary, and the tool cannot yet be recommended for routine clinical use. Validation in independent and prospective cohorts, ideally across multiple centers and diverse populations, will be essential to confirm its reproducibility and clinical utility.

First, the retrospective, single-center design may limit generalizability, as the findings reflect the characteristics of a hospital-based cohort in Northwest Romania. Second, the cross-sectional nature of the study precludes assessment of the score’s predictive value for future renal or cardiovascular outcomes. Third, the sample size reflects all eligible hospitalized patients, without an a priori power calculation, and some subgroups were very small (hyperuricemia-only, *n* = 4; no dyslipidemia/no hyperuricemia, *n* = 8). These small numbers reduce statistical power and increase the risk of unstable estimates; therefore, subgroup results should be interpreted with caution.

Fourth, the study population consisted exclusively of hospitalized patients with uncontrolled T2DM, who may represent a more severe subset compared with the general outpatient population. This could limit generalizability, and future studies should include ambulatory and community-based cohorts to enhance applicability. Fifth, several potential confounders—including dietary intake, alcohol consumption, physical activity, medication adherence, and inflammatory biomarkers—were not available in this retrospective dataset, which may have influenced the observed associations.

Finally, because long-term outcomes such as CKD progression, cardiovascular events, or mortality were not assessed, the prognostic value of RMRS remains uncertain. Future longitudinal and multicenter studies are needed to determine whether RMRS can reliably predict adverse outcomes and support clinical decision-making.

### 4.2. Global Application and Future Directions

The Renal–metabolic Risk Score (RMRS), constructed from routine laboratory tests (serum urea, TG/LDL ratio, and eGFR), has potential global applicability because it relies on simple, inexpensive, and universally available parameters. This makes the score particularly relevant in low-and middle-income countries, where access to advanced biomarkers or imaging is limited. In such settings, RMRS could provide a pragmatic approach for preliminary risk stratification, enabling earlier lifestyle counseling, pharmacological optimization, and referral to specialized care.

Future research should focus on validating RMRS in larger, multicenter, and outpatient cohorts, as well as on exploring its integration into electronic health records to facilitate early alerts for high-risk patients. Moreover, the addition of inflammatory biomarkers or genetic predispositions may further refine the model. Ultimately, prospective studies that include clinical outcomes will be essential to determine whether RMRS can improve patient management and inform global guideline development.

## 5. Conclusions

In patients with uncontrolled type 2 diabetes mellitus, the optimized Renal–metabolic Risk Score (RMRS)—integrating urea, TG/HDL ratio, and eGFR—demonstrated good discriminative performance for identifying the co-occurrence of dyslipidemia and hyperuricemia. Higher RMRS values were associated with a markedly increased prevalence of both conditions, showing a clear stepwise gradient across score quartiles. Given its reliance on routinely available laboratory parameters, RMRS offers a practical and cost-effective tool for early risk stratification in clinical settings. Prospective studies are warranted to validate its predictive value for long-term renal and cardiovascular outcomes and to assess its applicability across diverse populations.

## Figures and Tables

**Table 1:** Baseline demographic, clinical, and treatment characteristics of patients with uncontrolled type 2 diabetes mellitus according to the coexistence of dyslipidemia and hyperuricemia.

Parameters	Co-Occurrence	
	No	Yes
N	57	247
Age (years)	66.33 ± 11.51	66.90 ± 10.99
*p*-value	0.737	
Gender	Male 47.4%; Female 52.6%	Male 54.3%; Female 45.7%
*p*-value	0.429	
Provenance	Rural 56.1%; Urban 43.9%	Rural 55.9%; Urban 44.1%
*p*-value	1.000	
On uric acid-lowering therapy	Yes 8.8%; No 91.2%	Yes 15.4%; No 84.6%
*p*-value	0.280	
On lipid-lowering therapy	Yes 21.1%; No 78.9%	Yes 66.8%; No 33.2%
*p*-value	<0.001 *	
On antihypertensive therapy	Yes 78.9%; No 21.1%	Yes 89.9%; No 10.1%
*p*-value	0.040 *	

Data are presented as mean ± standard deviation (SD) for continuous variables and as percentages for categorical variables. *p*-values were obtained using independent-samples *t*-tests for continuous variables and chi-square tests for categorical variables. Significant results (*p* < 0.05) are marked with an asterisk (*).

**Table 2:** Baseline characteristics of study groups stratified by dyslipidemia and hyperuricemia status.

Group	N	Age	BMI	HbA1c	Systolic BP	Diastolic BP	Urea	Creatinine	eGFR	TG	LDL	HDL	Uric Acid
Both conditions	71	66.44	30.51	10.74	141.62	77.41	30.48	1.18	70.04	151.13	94.25	39.35	8.01
Dyslipidemia only	170	66.44	31.79	10.13	142.98	79.91	32.21	1.12	72.39	193.66	94.62	35.86	4.85
Hyperuricemia only	4	65	27.5	11.12	143	78.25	20.25	1.05	79.75	67.75	83	58.75	7.65
No dyslipidemia/No hyperuricemia	8	66.25	28.38	11.31	147.38	85	20.5	0.72	97.62	72.88	85.5	57	4.65

BMI—body mass index; HbA1c—glycated hemoglobin; BP—blood pressure; eGFR—estimated glomerular filtration rate; TG—triglycerides; LDL—low-density lipoprotein cholesterol; HDL—high-density lipoprotein cholesterol.

**Table 3:** Comparison of clinical and laboratory variables between patients with and without hyperuricemia, with effect sizes.

Variable	No Hyperuricemia (Mean ± SD)	Hyperuricemia (Mean ± SD)	*p*-Value	Effect Size
UREE (mg/dL)	20.04 ± 10.20	31.22 ± 20.74	2.70 × 10^−5^	Cohen’s d = 0.556 (moderate)
TRIGLICERID/LDL	1.75 ± 1.21	2.72 ± 6.19	2.91 × 10^−2^	Cohen’s d = 0.164 (small)
VARSTA (years)	65.12 ± 10.49	66.94 ± 11.13	0.415	Cohen’s d = 0.164 (small, NS)
HbA1c (%)	9.26 ± 2.87	9.66 ± 2.80	0.508	Cohen’s d = 0.143 (small, NS)
eGFR (mL/min/1.73)	77.76 ± 28.88	73.40 ± 25.91	0.472	Cohen’s d = –0.167 (small, NS)
SEX × Hyperuricemia	—	—	—	Cramer’s V = 0.053 (very weak)
Dyslipidemia × Hyperuricemia	—	—	—	Cramer’s V = 0.000 (none)

Data are presented as mean ± standard deviation (SD). *p*-values were obtained using independent-samples *t*-tests for continuous variables and chi-square tests for categorical variables. Effect sizes are expressed as Cohen’s d for continuous variables and Cramer’s V for categorical associations. NS—not significant. UREE—blood urea; LDL—low-density lipoprotein cholesterol; HbA1c—glycated hemoglobin; eGFR—estimated glomerular filtration rate; SD—standard deviation.

**Table 4:** RMRS distribution by co-occurrence status in uncontrolled T2DM.

Co-Occurrence Status	Mean	SD	Minimum	Maximum	*n*	*p*-Value
No (0)	6.82	5.22	2.29	18.30	182	1.2 × 10^−5^
Yes (1)	13.44	12.39	0.00	100.00	71	

**Table 5:** Distribution of Optimized Renal–Metabolic Risk Score.

Co-Occurrence Status	Median	IQR (Q1–Q3)
No (0)	10.00	5.15–15.61
Yes (1)	16.89	9.94–25.56

## Data Availability

The original data presented in the study are openly available in [repository name, e.g., FigShare] at 10.6084/m9.figshare.30336817.
